# Clinical and other specialty services offered by pharmacists in the community: the international arena and Israel

**DOI:** 10.1186/s13584-018-0251-y

**Published:** 2018-12-01

**Authors:** Eyal Schwartzberg, Joseph P. Nathan, Sivan Avron, Eli Marom

**Affiliations:** 1 LIU Pharmacy (Arnold & Marie Schwartz College of Pharmacy and Health Sciences), Brooklyn, NY USA; 2 School of Pharmacy, Ben-Gurion University of the Negev, Beer-Sheva, Israel; 3 Pharmaceutical and Enforcement Divisions, Ministry of Health, Jerusalem, Israel

**Keywords:** Adherence, Clinical services, Community pharmacy, Medications, Pharmacist, Rational drug consumption

## Abstract

The community pharmacy setting is a venue that is readily accessible to the public. In addition, it is staffed by a pharmacist, who is a healthcare provider, trained and capable of delivering comprehensive pharmaceutical care. As such, community pharmacists have a colossal opportunity to serve as key contributors to patients’ health by ensuring appropriate use of medications, preventing medication misadventures, identifying drug-therapy needs, as well as by being involved in disease management, screening, and prevention programs. This unique position gives the pharmacist the privilege and duty to serve patients in roles other than solely that of the stereotypical drug dispenser.

Worldwide, as well as in Israel, pharmacists already offer a variety of pharmaceutical services and tend to patients’ and the healthcare system’s needs. This article provides examples of professional, clinical or other specialty services offered by community pharmacists around the world and in Israel and describes these interventions as well as the evidence for their efficacy. Examples of such activities which were recently introduced to the Israeli pharmacy landscape due to legislative changes which expanded the pharmacist’s scope of practice include emergency supply of medications, pharmacists prescribing, and influenza vaccination. Despite the progress already made, further expansion of these opportunities is warranted but challenging. Independent prescribing, as practiced in the United Kingdom or collaborative drug therapy management programs, as practiced in the United States, expansion of vaccination programs, or wide-spread recognition and reimbursement for medication therapy management (MTM) programs are unrealized opportunities. Obstacles such as time constraints, lack of financial incentives, inadequate facilities and technology, and lack of professional buy-in, and suggested means for overcoming these challenges are also discussed.

**Electronic supplementary material:**

The online version of this article (10.1186/s13584-018-0251-y) contains supplementary material, which is available to authorized users.

## Background

The Israeli Pharmacist Ordinance [1] defines the term “pharmacist” and states that no person should be designated as a pharmacist unless properly licensed according to the directive of the Israeli Ministry of Health (MOH). In Israel, a person can become a pharmacist by earning a Bachelor in Pharmacy degree from 1 of the 2 Israeli schools of pharmacy (Ben Gurion University of the Negev [2] and The Hebrew University of Jerusalem [3]) and completing a required internship. Alternatively, pharmacists who were licensed abroad may attain licensure in Israel if they practiced in the original country of licensure for a period of time, as delineated in the Ordinance [4] or if they passed a state-administered examination in Israel. Those who seek to become “clinical specialists” can do so by completing the Doctorate Program in Clinical Pharmacy (Pharm.D.) at the Hebrew University of Jerusalem or a Master’s program in Community Clinical Pharmacy and Regulatory Management offered at the Ben-Gurion University of the Negev. Overall, those with advanced clinical degrees compose a small fraction of the total number of pharmacists practicing in Israel (< 3%). It is expected that the current undergraduate programs at the 2 schools of pharmacy will follow the curricula of pharmacy schools abroad to emphasize clinical content and practice.

Notably, in both schools of pharmacy in Israel, also within the undergraduate Bachelor’s program, students are educated/trained on consultation services, minor ailment management, and compounding activities. The graduate programs are aimed at providing advanced expertise and more extensive clinical training. The schools also offer continuing education courses for specialty training (e.g., medical cannabis, pharmaceutical consultation).

For further reading concerning the profession of pharmacy in Israel and educational requirements, the reader is referred to the article by Schwartzberg et al. [5]. In brief, traditionally, pharmacists in Israel focused on dispensing and compounding activities within pharmacies. However, since 2012, there have been extensive changes in the Pharmacists Ordinance and in regulations permitting pharmacists to expand their scope of practice, especially with regards to patient consultation and clinical interventions.

Unlike many other countries, Israel does not currently offer educational programs leading to certification as a “pharmacy technician” and the title is seldom seen in Israeli pharmacies. Instead, pharmacies rely on a team of pharmacists and pharmacy staff members (unlicensed personnel) who have no formal pharmacy education but assist in the logistical aspects of the pharmacy and not with medication dispensing.

It is well recognized within Israel [6–8], as well as worldwide [9–11], that the aforementioned description of a pharmacist does not adequately capture the healthcare service that he or she provides and that the pharmacist is an integral member of the healthcare team. As such, the pharmacist plays a pivotal role in the chain of delivery of healthcare services, assuring the patient’s safety and maintenance of quality of life. There are numerous examples of such non-traditional (non-compounding or dispensing) roles that pharmacists play within the scope of the delivery of healthcare services. In this article, we will provide some examples of non-traditional services delivered by pharmacists in the community setting worldwide and within Israel. In that context, we will analyze the evidence supporting such services as well as weaknesses that exist within community pharmacy, and we will conclude with recommendations for the future of community pharmacy practice in Israel.

## Main text

### Non-traditional/clinical services offered by pharmacists in the community – The international experience

Although the conventional definition of a pharmacist is likely to be similar around the world, professional requirements and the pharmacist’s practice skills diverge widely across countries [12]. While in some countries, such as Australia and India, pharmacists’ training and title (Bachelor in Pharmacy) match those requirements in Israel (or require an additional examination following graduation, as required in Canada), other countries have different training requirements and recognize pharmacists only after they acquire what Israel considers advanced degrees (Pharm.D. in the United States [US], or Master of Pharmacy [M.Pharm] in the United Kingdom [UK]). Moreover, health systems display vast differences across the world. Such variation further influences pharmacists’ training needs, skill sets, and service roles.

Melton and Lai reviewed the services provided by the community pharmacies in various countries [13]. The authors reported that, in general, pharmacists are providing more advanced services to patients and while patients are generally satisfied with the services they receive from pharmacists, their expectations are sometimes low and increase as exposure to advanced services increased. The authors concluded that pharmacists can have a greater impact on patient satisfaction through greater interpersonal skills than through the provision of new services; however, patients may be expanding their perceptions of how community pharmacists can be involved in their care beyond medication dispensing and counseling.

Perruadin et al. performed a systematic review to synthesize cost-effectiveness analyses on professional pharmacy services performed in Europe [14]. Twenty-one studies were included, conducted in the UK (*n* = 13), the Netherlands (*n* = 3), Spain (*n* = 2), Belgium (*n* = 1), France (*n* = 1) and Denmark (*n* = 1). The authors reported that professional services to enhance medicine safety (interprofessional meetings to reduce errors, *n* = 2) and access to medicines (minor ailment scheme, *n* = 1) were in favor of their cost-effectiveness in the UK context, but the evidence was not sufficient. Eleven studies assessed professional services to improve treatment outcomes of individual patients-such as pharmaceutical care services, medication review, educational and coaching program, disease support service, medicines management and telephone-based advisory for improving adherence. Findings were contradictory and did not lead to strong conclusion. Screening programs for different diseases showed robust positive results (*n* = 2) as well as smoking cessation services (*n* = 5) and should be considered to be more widely available in accordance with national context. The authors concluded that their review provides arguments for the implementation services aimed at improving public health through screening programs and smoking cessation service; but full economic evaluations are needed to support or refute the added value of other services.

Specific non-dispensing/unconventional activities performed by pharmacists in the community setting around the world have been implemented, assessed, and described in the literature. Within the UK there have been several programs [15] aimed at extending the role of the community pharmacist to include provision of services at time of changes in drug therapy, such as the “Help for HARRY discharge referral service”, where a referral is made to the patient’s chosen community pharmacy for advanced services (Medicines Use Review [MUR] or New Medicine Service [NMS]). A similar program (“Reablement Service”) has been offered on the Isle of Wight [16]. Patients identified as being at a higher risk of readmission are assessed by a hospital pharmacist prior to discharge and a referral to a community pharmacist is made and a home visit arranged. During such visits a full medication review is carried out by the pharmacist. Twigg et al. reported on another service offered in some community pharmacies in England [17]. This service, offered to patients over the age of 65 on 4 or more medications, involves pharmacist’s evaluation of the medications’ appropriateness, and discussions and consultations with the patients. Twigg et al. [18], describe a multidisciplinary team led by a community pharmacist providing MTM (comprehensive medication review and self-management education) to geriatric patients (65+), high-risk, diabetic Medicare beneficiaries in the US. In another study [19] conducted at an independent community pharmacy in the Midwest US, patients whose proportion of days covered (PDC; a measure of the percent of time that patients have their medication on hand, available for use) for their oral anti-diabetic medications was less than 80% were counseled by phone by a pharmacist. Abughosh et al. [20] described a program involving a brief pharmacist telephone intervention to identify adherence barriers and improve adherence to certain medication classes in non-adherent patients with comorbid hypertension (HTN) and diabetes mellitus (DM) who were enrolled in a Medicare Advantage plan in Texas. Kovačević et al. described a study [21] involving Serbian community pharmacies, in which pharmacists delivered a 30-min counseling session on asthma. Other single-intervention studies among asthmatics have been conducted in several countries (Schulz et al. in Germany [22], García-Cárdenas et al. in Spain [23], and Wong et al. in Malaysia [24]). In a study by Närhi et al. [25], 31 patients suffering from unstable asthma consulted with physicians, nurses, and at least once every 3 months with their community-pharmacists over the period of 1 year. A study by Stuurman-Bieze et al. [26] included 1002 patients initiating lipid lowering therapy at 9 Dutch community pharmacies. The investigators utilized and assessed the effectiveness of a proactive pharmaceutical care intervention (Medication Monitoring and Optimization; MeMO) plan, that continued for a year, on the discontinuation rate and patient adherence as compared to a historical control group. In a study by Holdford and Inocencio [27], conducted in rural Midwestern US, the investigators examined the appointment-based medication synchronization (ABMS) method on medication adherence and persistence with chronic medications. In the UK, pharmacists at 192 community pharmacies were trained to provide a first line, urgent care service for minor ailments under a campaign called “The Pharmacy First” [28]. Immunization by pharmacists has also gained in popularity around the world. According to a 2016 report by the International Pharmaceutical Federation [29], as of 2016, 13 countries authorized pharmacists to administer vaccines. Disease screening programs are available in community pharmacies in several countries. In 2012–2013 a pilot program in the UK offered a screening service in community pharmacies for early detection of COPD [30]. Likewise, in many European countries, community pharmacists screen for high blood pressure, BMI and blood glucose and cholesterol levels [31]. Also according to the report, bowel cancer screening programs are provided through pharmacies in Italy, Spain, and Switzerland. Examples of other chronic disease management programs and educational programs conducted in some European community pharmacies include diabetes management, asthma management, hypertension management, and smoking cessation. A more detailed description of all of the aforementioned activities is available in the Additional file 1.

### Pharmaceutical services currently authorized in the community setting in Israel

In Israel, as defined in the “Patient’s Rights” document, a pharmacist is considered a healthcare provider [7]. As of 2015, there were approximately 1885 pharmacies in Israel [32]; 40% of them owned and operated by health maintenance organizations (HMOs), 45% privately owned, and 15% belonging to big health and beauty retailers. Community pharmacies are open for most of the day and municipalities tend to designate at least 1 pharmacy that provides emergency services on weekends and during “off” hours. Because of this, community pharmacists are fairly readily available to provide health services. Thus, visiting a pharmacy may be more convenient for the patient than scheduling an appointment with a physician. Notably, only a physician is allowed to diagnose medical conditions and prescribe all medications for labeled and unlabeled uses. In this section, we will review the main services the Israeli pharmacist currently offers or is authorized to offer in the community setting.

#### Medication advice and guidance

The MOH has established regulations defining the basic scope of pharmaceutical services in Israel [6, 7]. These regulations declare the pharmacist to be a healthcare professional who has a responsibility for ensuring proper pharmaceutical care. As these relate to the management of minor ailments, the pharmacist evaluates and triages a patient presenting with a medical complaint and determines whether the situation warrants referral to a physician or whether it can be self-managed, for example with over-the-counter (OTC) medications. In the latter case, the pharmacist also has the duty to determine the appropriate OTC for the given patient and situation and educate the patient about the proper use of the recommended treatment. These regulations were set in the Pharmacy Ordinance and Regulation 112, which describes in detail the procedures that the pharmacist must follows when interviewing (obtaining necessary information from a patient) and counseling a patient in the community setting [6]. The aforementioned process is obligatory for all pharmacists in the community setting, and thus must be performed on routine basis.

With respect to the management of chronic illnesses, MOH regulations [33] specify that all chronically-medicated patients must be evaluated when prescribed a new medication as well as yearly by a trained physician or by a clinically-trained pharmacist (since 2016). Moreover, the pharmacist has the right to provide consultation services. This includes a pharmacist-initiated, professional counseling session, during which the pharmacist reviews the patient’s medication (and supplement) list and blood test results and measures other biometric parameters (blood pressure, weight, Hgb A1C and glucose, etc.) using validated consultation tools [34–36]. Throughout the counseling session, the pharmacist advises the patient about a healthy lifestyle and provides information about medications taken, recommended changes in medications, and the importance of treatment adherence.

In Israel, HMOs have to meet certain requirements in order to receive governmental grants for developing new services. The MOH has published financial support requirements for the 4 HMOs in Israel (Clalit Health Services, Meuhedet Health Services, Maccabi Healthcare Services, and Leumit Health Services) recommending that active pharmaceutical consultation be performed for at least 3% [37] of the chronic patients^1^ using polypharmacy. Three of the HMOs have responded to the requirement, receiving in total close to 4 million NIS [38]. This is indicative of the MOH’s recognition of and interest in pharmaceutical interventions. To date, these tasks are performed by pharmacists in a few pharmacies in the community setting, including HMO-owned pharmacies as well as chain pharmacies. According to data on file with the MOH, the HMOs currently providing such services include Clalit, Maccabi, and Leumit.

#### Emergency supply of medications

A 2016 MOH regulation “Dispensing medication without prescription for an immediate critical need” [8] has expanded the pharmacist’s authority to include the dispensing of medications without a prescription under certain conditions. With 2 years of pharmacy practice experience (or the possession of a degree in clinical pharmacy), a pharmacist is entitled to dispense previously prescribed medications without a prescription, based on his or her clinical judgment of the patient’s situation and importance of an uninterrupted drug therapy in the patient. The pharmacist is to conduct a comprehensive questioning of the patient’s condition and ascertain the reason that he or she was unable to obtain a prescription, ensure that the medication is not in the “controlled or psychotropic drugs” list, and determine whether dispensing the medication is for the patient’s benefit while maintaining his or her safety. Further restrictions that may apply are delineated in the regulation. This service is currently permitted in all community pharmacies, subject to the discretion of the pharmacist.

#### Pharmacists prescribing: Dependent and independent prescribing

Dependent prescribing [39] is defined as the authority of some pharmacists (i.e. those with the required 5 years of experience or a degree in clinical pharmacy, and with proper training) to re-prescribe/extend a prescription for medications appearing on a designated list (list in [39] Additional file 1). This authority is limited to prior existing conditions and restricted to when the pharmacist’s prescription does not extend beyond 6 months from the physician’s last prescription and to situations when the physician met with the patient within the last 9 months. When all these conditions are met, the pharmacist is required to ask for the patient’s consent and review the patient’s medications and health status in order to conclude whether the re-prescribing is warranted. Such decisions requires full medical access, and therefore the pharmacist is granted medical information access from the patient as well as access to medical details available on the patient’s computerized medical records. Because of these requirements, this is usually performed only in HMO-based community pharmacies where the pharmacist has access to all the required information.

The authority of the trained pharmacist was recently expanded to independent prescribing, whereby a pharmacist who holds the authority to prescribe (as described above) can now prescribe, without a physician’s consent 21 designated prescription-only medications for specific conditions ([39] Additional file 1). This type of service was recently approved by the Israeli parliament and was added into the pharmacy regulation. Furthermore, several hundred pharmacists have completed the required academic component in order to implement this service. However, although approved legally, this type of service has yet to be implemented by the HMOs.

#### Compounding services

In many community pharmacies (private, chains, and HMO-based) pharmacists also compound medications when extemporaneous compounding is necessary. This is mainly done for the pediatric population or in other situations when the required dose or dosage form is not commercially available. In addition, compounding is common for drugs with a short shelf life, when treating rare diseases, and/or in instances of drug shortages. In certain pharmacies, homeopathic compounding is also offered. The conditions which qualify for such services are defined and published in procedures 10, 132 and 135 [40–42]. This service is authorized in all pharmacy settings. However, because of logistical and medical reasons, pharmacies may elect not to provide such services. In such instances, the pharmacist needs to refer the patient within a given time frame to one of several approved compounding pharmacy facilities that are available within Israel, in order to provide the medication in a timely fashion.

#### Influenza vaccination

Starting in 2017 [43], appropriately-trained community pharmacists are authorized to administer influenza vaccines to suitable adult patients during the influenza season. This allows more patients to have easy access to the vaccines and is expected to increase percentage of the immunized population. This expansion in the pharmacist’s scope of practice is also expected to enhance the recognition of the pharmacist as a healthcare provider. To date, although several pharmacists are trained and authorized to administer the influenza vaccine, this service has only been implemented in 1 privately-owned pharmacy. The Ministry is working to facilitate the expansion of these services to other pharmacies.

#### Biometric testing

In designated pharmacies, upon request from the patient, pharmacists also offer biometric testing services, including measuring blood glucose levels, blood pressure, and BMI [7]. The service may be offered by pharmacist who have been in practice for at least 2 years and who underwent training in the proper usage of the necessary equipment. Notably, the pharmacist cannot make a diagnosis based on the results but rather refer the patient for proper care by a physician. This service is authorized in all community pharmacy settings and has been implemented in some, based on the pharmacist’s discretion.

#### Patient access to pharmaceuticals

The MOH allows drug product delivery by messenger directly from a pharmacy to the consumer or as an online purchase [44]. This service provides access to pharmaceuticals to community-dwelling patients who might not be able to come to the pharmacy to receive the medication. This, in turn, may raise the medication-adherence rates in this population. Such messenger service requires adequate transportation conditions that ensure that all required storage conditions are met throughout the delivery process as well as a method to document receipt of the prescription and drug product. In addition, designated pharmacies also offer access to drugs that are unregistered in Israel. This applies when such drugs are specially ordered by a physician for a specific patient and condition. This service is approved under the pharmacy legislation Clause 29(c) [45]. These services are authorized in all pharmacies and have been implemented in some.

#### HMO-initiated projects

Due to the low ratio of clinically-trained pharmacists to the population in community settings, HMO management tends to utilize their few clinical pharmacists “behind closed doors”. Thus, clinical pharmacists are usually based in an office where they are commonly involved in drug policy and protocol development. When they review patients’ medical records, communication concerning necessary interventions takes place directly with the physicians. Direct communication with the patient is usually not a part of this process. Anecdotally, this model is primarily seen in Maccabi, while in Clalit and Leumit, in-house training of pharmacists who do not possess formal credentials in clinical services, produced pharmacists who provide patient consultation services for their clients.

A six-month prospective study involving 588 diabetic patients insured by the Maccabi HMO with HbA1c ≥ 8.5 was conducted by Lomnicky et al. [46]. In this study, 270 of the patients received pharmaceutical intervention and the others were assigned to the control group. The medication adherence of the patients receiving pharmaceutical intervention was shown to be 13% higher than those in the control arm. There was a significant (− 1.02 ± 0.52, *p* = 0.05) decrease in HbA1c among counseled patients of low socio-economic status but not in the similar patients in the control group. This project demonstrated the potential influence that pharmaceutical intervention may have, particularly in a low socio-economic population.

A survey by Brammli-Greenberg et al. [47] was administered at 11 clinics and pharmacies of the 4 HMOs across the country. A total of 260 people (50 “policy makers” and 210 patients of the HMOs) were interviewed about their satisfaction with pharmaceutical consultation. The researchers found that the patients believed that policy makers should consider community consultation service to be provided by HMOs and that it should be under the HMOs’ supervision. However, the interviewees suggested that the state needs to motivate the HMO to implement such services. Other noteworthy findings were that the pharmacist should work in collaboration with the physician and that the consultation be provided to the patient, that the preferred communication method should be face-to-face, and that the consultation program should be provided on a long-term basis. Most of the insured patients felt that the consultation should be provided by their physician, not a pharmacist.

Another study conducted by Triki [48] in the Maccabi HMO evaluated the influence of pharmacists’ interventions, in the form of communication with the patients’ physicians, on diabetic patients’ LDL levels in comparison to a control group which did not receive pharmacists’ interventions. The study found that in the intervention group, 67% of the patients achieved a target LDL value over the year, compared with 54% of the patients in the control group. In addition, the patients in the intervention group reached a target LDL level about 3 months earlier than the patients in the control group. This study demonstrated the pharmacist’s positive influence even without direct patient interaction, and could justify the current method of utilization of clinical pharmacists by most HMOs.

Another study by Triki et al. [49] examined a pharmacist intervention method that involved distribution of information leaflets on the proper use of inhaled steroids to physicians, nurses, and patients. The leaflets were aimed at providing instructions on when to use steroid inhalers. The use of inhaled steroids was examined over a period of a year and compared the “before” and “after” usage. The researchers found that the intervention resulted in a decrease of approximately 53% in the removal of these drugs from the nurses’ rooms in the Shfela District, which represents an estimated decreased expenditure of approximately NIS 450,000. In addition, there was a general decrease of 48% in the consumption of inhaled steroids compared with the previous year. This study demonstrated the need for educating the healthcare staff in the community setting and the potential health and financial benefits such programs can offer to the patient and to the HMO.

## Discussion

Pharmaceutical care has been defined as “patient-centered, outcomes oriented pharmacy practice that requires the pharmacist to work in concert with the patient and the patient’s other healthcare providers to promote health, to prevent disease, and to assess, monitor, initiate, and modify medication use to assure that drug therapy regimens are safe and effective” [50]. Table 1 summarizes how elements of pharmaceutical care process were implemented into Israel’s community pharmacy practice over the years.

**Table 1:** Timeline of Regulation of Pharmacy Services in Israel

Service	Regulation No.	Year of addition into pharmacy regulation	Status as of 2018
Drug dispensing	The Pharmacists’ Ordinance [1]	1981 (The Pharmacist’s Ordinance was in effect prior to the establishment of Israel in 1948.)	Implemented
Dispensing of unregistered drugs based on individual needs	The Pharmacists’ Ordinance; regulation 29, clause C [48]	2007	Authorized in all pharmacies; practiced in most pharmacies
Medication advice and guidance	Regulation no. 112 and 113 [6, 7]	2013	Authorized and practiced in all pharmacies
Biometric testing: Measuring blood glucose levels, blood pressure, and body mass index (BMI)	Regulation no. 113 [7]	2013	Authorized in all pharmacies; practiced in many community pharmacies, especially chain pharmacies
Dependent and independent prescribing	The Pharmacist Ordinance addition, Pharmacist Prescription [1] [8]	2014	Authorized in all pharmacies contracted with an HMO; not yet practiced
Extemporaneous compounding	132 and 135 [40] [42]	2000, 2014	Simple compounding authorized and practiced in all pharmacies; complex/high risk compounding authorized and practiced in some pharmacies
Messenger drug delivery	128 [44]	2014	Authorized in all pharmacies practiced in few pharmacies
Emergency supply of medications	154 [8]	2016	Authorized and practiced in many pharmacies (mostly privately-owned)
Influenza vaccination	160 [43]	2017	Authorized in all pharmacies; implemented in 1 pharmacy

As evident from the Table 1 until about 15 years ago, pharmaceutical services in Israel primarily involved the dispensing of medications. However, over the past several years, additional services were approved by the legislature. These include pharmacists’ prescribing, vaccination, emergency supply of medication, and medication-use review, as well as elements of patient monitoring authority. Despite the progress already made, the need for additional progress remains. Although pharmacists in Israel are trained comprehensively to perform many non-dispensing pharmaceutical care tasks and are authorized to perform such activities, challenges exist that may hold back the expansion in the pharmacist’s role to encompass the broad definition of pharmaceutical care. Such challenges include the followings:

### Time factor

Many pharmacies are understaffed, necessitating the pharmacist to provide services single-handedly. This may prevent the pharmacist from providing pharmaceutical care per the definition noted above. The authors suggest that in order to overcome the time obstacle, automation needs to be introduced into pharmacies to allow the pharmacist’s time to be utilized for clinical and consultation activities. Much like many other industries, the pharmaceutical profession is slowly becoming integrated with technology. To date, dispensing robots have already been implemented in 2 medium-sized hospitals in Israel [51, 52] and in key HMO-based community pharmacies in the country. These robots, dispense medications without errors and provide higher levels of efficiency than ever before.

Despite the advancements made and the expectation that such automation will become even more common in Israel, with the MOH already issuing regulations to standardize the process [53], relative to many other countries, Israel is somewhat behind in incorporating such technologies. Throughout the world, robots have been in place in community pharmacies, allowing the robots to save time for the pharmacy staff and for the patients while performing with minimal errors. The robots can dispense and prepare medications in bags for patients and provide alerts about expiration dates and storage requirements [54].

Automation is expected to have 2 main results. First, the pharmacist will have more time to offer more advanced pharmaceutical services to patients. Second, automation should allow pharmacy managers to employ fewer staff members and in the long run, reduce the overall operational costs of the pharmacy.

The implementation process requires a comprehensive change of concept as well as organizational changes. Furthermore, a financial investment will be necessary as well.

Other strategies to improve time efficiency at the pharmacy may include programs such as the ABM [27]. This approach is believed to improve patients’ medication adherence, build efficiency into the pharmacy workflow, and allow for managing potential issues before the patients arrives at the pharmacy to pick up their medications. Developing pharmacy technicians/assistants training programs may be useful for supplying community pharmacies with properly-trained individuals and this, in turn, can lessen the pharmacist’s workload and free him or her to offer services.

### Remuneration

Economic pressure resulting from pharmacists being remunerated based on a fee-per-item and not based on professional services is likely to contribute to suboptimal pharmaceutical care. This approach, which has been used for decades by the HMOs and accepted by pharmacists, serves as an obstacle for developing professional services which are not dispensing-based. Pharmacists’ remuneration should include a fee for professional service instead of being based on dispensing fees only. If pharmacists were to charge patients privately for such services, it is likely that such a program would not be overly successful, especially since standard fees are not in place.

### Facilities

Currently many pharmacies do not have a private consultation room. This in turn hinders the ability to provide professional services which require a level of privacy and not suitable for over-the-counter consultation. As such, the Pharmacist Ordinance [1] was changed to mandate that such a room exists. In addition, pharmacist could offer a consultation service using technological accessories (such as telemedicine technologies or a video-chat service offered today in Israel by some physicians and HMOs).

Once the time and facilities challenges are resolved, pharmacists may concentrate on patients’ consultation, using the patient’s medical record, as well as on health education. Research by Zwaenepoel et al. [55] has shown that education on a patient’s health condition, offered at a community pharmacy can have a triple positive effect. First, it would increase cooperation between the patient and the healthcare staff. Second, it may increase the treatment success rate, as the education raises awareness and adherence. Lastly, a patient’s understanding of his or her condition over time decrease patient’s expenditures on medical treatment, thereby saving money to the patient as well as the health system.

### Technology

In order for the pharmacist to be able to comprehensively assess the patient and his or her pharmaceutical care needs, the pharmacist needs to have access to data about the patient. These include basic demographic information as well as laboratory data and any other relevant information. Without having such information, it may be difficult for the pharmacist to adequately review the patient’s medications and determine their appropriateness. Likewise, such information is necessary when considering the use of other non-drug products such as dietary supplements and other alternative remedies. Currently, pharmacies owned or contracted by the HMO generally have access to the patients’ medication history and some HMOs have implemented or are considering the implementation of computerized decision support systems for identification of drug interactions. It is expected that once prescribing services are implemented, the pharmacists will have access to other medical data, such as laboratory results, as mandated by the regulation. Full integration of pharmacy computer systems with the HMO’s or with other medical data is challenging due to financial constraints, privacy restrictions, and lack of realization by top HMO management of the benefits that such technology may offer. Until computer systems are fully integrated to provide the pharmacist with a full picture of the patient’s conditions and needs, pharmacists are urged to utilize whichever tools and information they do have (e.g., blood glucose monitoring, BP, BMI) and their own professional acquaintance with the patients they service (e.g., through questioning or otherwise), in order to build a patient profile that will instrumental for delivering more comprehensive services.

### Professional reputation

Studies have shown that in Europe as well as in Israel, patients prefer to receive counseling regarding medical treatments from physicians; this is true even when the subject of the consultation is strictly pharmaceutical. While this may be clinically appropriate in some situations, in others it probably results in suboptimal utilization of the physician’s time and of HMO’s financial resources [56, 57]. Thus, it is imperative that the public’s perception of the pharmacist changes so that the pharmacist be recognized as an integral member of the healthcare team and as a professional who is capable and readily available to deliver some of the services that traditionally were provided in physicians’ offices only.

In Israel, as in many other countries, pharmacists are often associated with retail activities rather than with clinical and consulting services. The reasons for this may be attributed to 2 main reasons. First, the public may not be aware of the educational requirements for becoming a pharmacist and therefore they may under-appreciate the extensive knowledge, skills, and capabilities that pharmacists possess. Second, pharmacists must be recognized by other members of the healthcare team as healthcare providers who are capable and ready to deliver pharmaceutical care in a collaborative manner while maintaining each profession’s unique authority and scope of professional practice and capabilities. This is especially important in an era when interprofessional education and collaboration are emphasized as means to improve patient outcomes.

It is important to note, though, that in order for the public’s and the healthcare community’s perception to change, the pharmacist’s mindset concerning his or her profession needs to change as well. Pharmacists’ own desire to expand their role and the recognition that they can achieve that goal, must be the driving force for many of these changes.

### Other factors

One of the main factors impeding the development and adoption of a broader scope of practice for pharmacist in Israel has been the opposition of the medical profession, specifically the Israeli Medical Association (IMA). From the very early days of the amendments of the pharmacist Ordinance, allowing pharmacist to prescribe, and throughout the enactment of the pharmacy regulation, IMA expressed their concerns with regard to the above legislations [58–60]. Reasons varied from lack of sufficient education and pharmacists’ knowledge to engage in clinical services, harming the public’s health, presenting dangerous situation for patients’ safety and efficacy of medical treatment, as well as jeopardizing patient’s privacy. Position papers were published prior to every discussion in the Knessest. However, although slowing the legislative process, the legislations were passed by the legislators.

As for the HMOs, their position was positive and allowed for the above enactments; however, due to logistical and financial reasons, as described above, the implementation of pharmacist prescribing regulation, as well as other clinical services, has been slow and it is still in infancy stages.

Although there is a long way to go to fully implement and reimburse pharmacists for clinical and professional services in the community setting, it is encouraging to see that the legal foundation was laid and supported by the legislators as well as by key opinion leaders within the HMOs and the MOH.

### Targeted efforts

As described earlier, pharmacists’ interventions may be of great benefit to patients and to the healthcare system as a whole. However, in order to strategically implement such projects in a fashion that will be most effective and efficient from the patients’, pharmacists’, health plans’, physicians’, and the system’s perspective, the authors suggest that pharmaceutical interventions be aimed at the following groups of patients.

#### Patients receiving polypharmacy

Patients receiving 5 or more medications concomitantly [61] are prone to ADRs, drug interactions, and low adherence. A comprehensive evaluation and consultation service by the pharmacist, can have a positive impact on the safety, financial state, and quality of life of these patients [17]. A phone-interview conducted by Vaknin et al. [61] questioned 200 pharmacists about whether they are familiar with their patients who receive polypharmacy. The results of the interviews revealed that about 75% of the pharmacists were familiar with their patients who were exposed to polypharmacy when they repeatedly visited the same pharmacy. Although most of the pharmacists had received advanced training on medication management, which would have been particularly useful for managing such patients, only 50% of the surveyed pharmacists actively practiced it with their patients. Reasons noted for this were unsuitable environments (shortage in manpower and unsuitable infrastructures), lack of access to patient medication files, and lack of patient cooperation. Therefore, it appears that there is a large unmet need for medication management services in patients with polypharmacy.

#### Chronic patients

According to the MOH [62], a chronic patient is a patient treated for certain diseases, one treated with certain drugs, or any patient taking a specific prescription drug for at least 6 consecutive months. Within this population, it is possible to identify key “high risk” sub-populations which would benefit the most from a more comprehensive pharmaceutical care. These include:

- *Patients taking chronic medications*: Including cardiovascular drugs such as ACEIs/ ARBs, thiazide diuretics, beta blockers, dihydropyridine calcium channel blockers, statins, anti-diabetic agents, such as metformin and sulfonylureas, and anti-depressants, such as selective serotonin reuptake inhibitors (SSRIs)/serotonin norepinephrine reuptake inhibitors (SNRIs).
- *Patients with diabetes or pre-diabetes*: Pharmaceutical intervention which may include blood glucose monitoring, hemoglobin A1C monitoring, as well as educational sessions, may assist in controlling the patient’s condition. A suggested list of qualified patients for such a service could be determined with a specialized staff in order to screen and identify high-risk patients.
- *Patients with asthma or COPD:* Patients with asthma step 5 disease^2^ [63] and above and patients with COPD exacerbations^3^ [64] are often treated with oral steroids, which require special attention to ensure a rational and safe use of the medications. Pharmaceutical monitoring would help reduce the severity of the disease in asthma and prevent illness progression and exacerbations in both asthma and COPD. In addition, patients should receive proper instructions about the use of their inhalers at the time of diagnosis and medication prescribing.

In addition, the authors recommend targeting other high-risk populations that may benefit from singular or periodical pharmaceutical intervention. These include patients released from hospitals to the community, since they are often in need of a medication reconciliation service, patients moving between different healthcare systems (e.g., Israel Defense Forces (IDF), different HMOs), and other special situations when a need is identified and patients are referred by the physician for clinical pharmaceutical consultation services .

## Conclusion

It appears that community pharmacy services in Israel have made great strides over the years. However, when comparing the community pharmacy landscape in Israel with that seen around the world, it appears that the Israeli community pharmacy scene falls short of its potential when it comes to the provision of comprehensive pharmaceutical care. Unfortunately, community pharmacies in Israel seldom offer and conduct an in-depth medication usage review or offer other disease screening, management, or preventative services. Instead, community pharmacists concentrate mainly on medication dispensing and on pharmaceutical services related to OTC products. This may be due to time limitations, financial constraints, inadequate facilities, lack of health informatics technologies, and lack of a buy-in from the pharmacy community, other healthcare professionals, and from the public.

These aforementioned challenges can and should be overcome. Whenever possible and appropriate, pharmacies should be equipped with robots which should allow for the pharmacist’s time to be freed for clinical services. The buds of this change are now blooming and the first dispensing robots were recently incorporated by certain HMOs. Similarly, utilizing non-pharmacists assistants may produce the same desired effect. This may be achieved by implementing a pharmacy in house training program to supply community pharmacies with properly-trained assistants and subsequently freeing some of the pharmacist’s time for other services. The government should consider providing financial support for advanced pharmaceutical services as was done for some of the previously programs in the UK. Furthermore, the remuneration model currently implemented by the HMOs in Israel should be changed from dispensing fee per-item to a fee-for-service model, thereby encouraging pharmacists to offer documentable services, such as walk-in clinics, health screening and consultations, and vaccination services. Pharmacies should include a designated consultation room to allow for patient privacy. Steps towards this have already been taken as evident in the recent changes introduced by legislation to the Pharmacist Ordinance. Key to the successful provision of efficient and accurate consultation service is the ability to access relevant patient information. Thus, connecting the pharmacist with patient’s health records is essential for the delivery comprehensive pharmaceutical services. Finally, gaining recognition as a healthcare provider is challenging but it must begin with advocacy within the profession itself. Pharmacists should desire to explore and implement new service options. If successful, such programs can serve as the evidence necessary for a buy-in from other healthcare professionals and from the public. This change may need to begin by adapting the undergraduate and graduate pharmacy curricula in Israel to address advanced professional courses and needs to continue post-graduation in the form of continuing education courses.

Based on the evidence presented, changes to the Israeli community pharmacy model are needed. These changes should be focused on moving from product-oriented services to patient-oriented services. The authors believe that such changes are possible and have the potential of improving health outcomes for the individual patient as well as improving the healthcare system as a whole.

## Additional file

Additional file 1: Examples of Clinical and other Specialty Services Provided by Pharmacists in the Community Internationally. (DOCX 35 kb)

## Abbreviations

ABM: Appointment Based Model
ABMS: appointment-based medication synchronization
ADE: Adverse drug effects
ADR: Adverse drug reactions
BMI: Body mass index
COPD: Chronic obstructive pulmonary disease
CVD: Cardiovascular disease
ER: Emergency room
FIP: International Pharmaceutical Federations
HMOs: Health Maintenance Organizations
IDF: Israel Defense Force
IMA: Israeli Medical Association
M. Pharm: Master of pharmacy
MAS: Minor ailment service
MeMO: Medication Monitoring and Optimization
MOH: Ministry of Health
MTM: Medication Therapy Management
MUR: Medicines Use Review
NMS: New Medicine Service
NSAIDs: Non-steroidal anti-inflammatory drugs
OTC: Over-the-counter
PDC: Proportion of days covered
PGEU: Pharmaceutical Group of European Union
Pharm D: Doctor of pharmacy
PPIs: Proton pump inhibitors
RCT: Randomized controlled trials
SNRIs: Serotonin Norepinephrine Reuptake Inhibitors
SSRIs: Selective Serotonin Reuptake Inhibitors
UK: United Kingdom
US: United States

## Footnotes

1 According to the Health Ministry [38], a chronic patient is defined either by the diagnosis of a chronic illness or by the medication the patient is taking; the medication can be any medication from a given list, or any other medication prescribed for over six months.

2 According to 2007 NHLBI Guidelines for the Diagnosis and Treatment of Asthma Expert Panel.

3 COPD stages by the GOLD system, stage III defined as: FEV1/FVC ratio less than 70%, FEV1 30% to less than 50% predicted value, may have chronic symptoms and treated among other treatments with corticosteroids.

## References

[1] State of Israel. Ministry of Health. Pharmacists ordinance [new version] (May 2016). http://www.health.gov.il/LegislationLibrary/Rokhut23_20161007.pdf. Accessed 29 Jan 2018.

[2] Ben Gurion University of the Negev, Bachelor degree program, School of Pharmacy, The Faculty of Medicine, Ben Gurion University of the Negev. http://in.bgu.ac.il/welcome/Pages/degree_1/Pharmacy_degree_1.aspx. Accessed 29 Jan 2018.

[3] The Hebrew University of Jerusalem, Offered programs. School of Pharmacy. The Faculty of Medicine, The Hebrew University of Jerusalem. http://info.huji.ac.il/bachelor/School-Of-Pharmacy Accessed 29 Jan 2018.

[4] State of Israel. Knesset. Amendment (26) to Pharmacists Ordinance. http://fs.knesset.gov.il//20/law/20_lsr_366976.pdf. Accessed 29 Jan 2018.

[5] Schwartzberg E, Nathan JP, Rosen B, Marom E (2018). Pharmacy in Israel. Am J Health Syst Pharm.

[6] State of Israel. Ministry of Health. Regulation no. 112 - Pharmaceutical interview and counseling when dispensing medicinal products in a pharmacy and/or medications room in the community. https://www.health.gov.il/hozer/DR_112.pdf Accessed 29 Jan 2018. [Hebrew].

[7] State of Israel. Ministry of Health. Pharmaceutical Division. Regulation no. 113 Initiated pharmaceutical counseling, drug information and monitoring by a pharmacists in community pharmacy and medical centers. https://www.health.gov.il/hozer/DR_113.pdf. Accessed 29 Jan 2017. [Hebrew].

[8] State of Israel. Ministry of Health. Regulation no. 154 - Drug distribution without prescription conducted by a pharmacists for an immediate need. https://www.health.gov.il/hozer/DR_154.pdf Accessed 29 Jan 2017. [Hebrew].

[9] Foppe van Mil JW, Schulz MA (2006). Review of pharmaceutical care in community pharmacy in Europe. Harvard Health Policy Review.

[10] Shord SS, Schwinghammer TL, Badowski M, Banderas J, Burton ME, Chapleau CA (2013). Desired professional development pathways for clinical pharmacists. Pharmacotherapy.

[11] Noyce PR (2007). Providing patient care through community pharmacies in the UK: policy, practice, and research. Ann Pharmacother.

[12] Pharmaceutical Group of European Union. Overview of Community Pharmacy Services in Europe (PGEU). https://www.oecd.org/els/health-systems/Item-2b-Overview-Community-Pharmacy-Services-Svarcaite%20.pdf. Accessed 22 Jan 2018.

[13] Melton BL, Lai Z (2017). Review of community pharmacy services: what is being performed, and where are the opportunities for improvement?. Integr Pharm Res Pract.

[14] Perraudin C, Bugnon O, Pelletier-Fleury N (2016). Expanding professional pharmacy services in European community setting: is it cost-effective? A systematic review for health policy considerations. Health Policy.

[15] NHS England. Quick Guide. Extending the Role of Community Pharmacy in Urgent Care. https://www.england.nhs.uk/commissioning/wp-content/uploads/sites/12/2015/11/quick-guid-comm-pharm-urgent-care.pdf. Accessed 23 Jan 2018

[16] NHS Isle of Wight, Getting medicines right in hospital and at home, http://www.iow.nhs.uk/default.aspx.locid-02gnew08v.Lang-EN.htm. Accessed 23 Jan 2018.

[17] Twigg MJ, Wright D, Barton GR, Thornley T, Kerr C (2015). The four or more medicines (FOMM) support service: results from an evaluation of a new community pharmacy service aimed at over-65s. Int J Pharm Pract.

[18] Twigg G, Motsko J, Thomas J, David T (2017). Pharmacist-managed diabetes center interventions ensure quality and safety in elderly patients. Consult Pharm.

[19] Singleton J, Veach S, Catney C, Witry M (2017). Analysis of a community pharmacy intervention to improve low adherence rates to oral diabetes medications. Pharmacy.

[20] Abughosh SM, Wang X, Serna O, Henges C, Masilamani S, Essien EJ (2016). A pharmacist telephone intervention to identify adherence barriers and improve adherence among nonadherent patients with comorbid hypertension and diabetes in a Medicare advantage plan. J Manag Care Spec Pharm.

[21] Kovačević Milena, Ćulafić Milica, Jovanović Marija, Vučićević Katarina, Kovačević Sandra Vezmar, Miljković Branislava (2018). Impact of community pharmacists' interventions on asthma self-management care. Research in Social and Administrative Pharmacy.

[22] Schulz M, Verheyen F, Mühlig S, Müller JM, Mühlbauer K, Knop-Schneickert E (2001). Pharmaceutical care services for asthma patients: a controlled intervention study. J Clin Pharmacol.

[23] García-Cárdenas V, Sabater-Hernández D, Kenny P, Martínez-Martínez F, Faus MJ, Benrimoj SI (2013). Effect of a pharmacist intervention on asthma control. A cluster randomized trial. Respir Med.

[24] Wong LY, Chua SS, Husin AR, Arshad H (2017). A pharmacy management service for adults with asthma: a cluster randomized controlled trial. Fam Pract.

[25] Närhi U, Airaksinen M, Tanskanen P, Erlund H (2000). Therapeutic outcomes monitoring by community pharmacists for improving clinical outcomes in asthma. J Clin Pharm Ther.

[26] Stuurman-Bieze AGG, Hiddink EG, van Boven JFM, Vegter S (2013). Proactive pharmaceutical care interventions improve patients’ adherence to lipid-lowering medication. Ann Pharmacother.

[27] Holdford DA, Inocencio TJ (2013). Adherence and persistence associated with an appointment-based medication synchronization program. J Am Pharm Assoc.

[28] NHS England. Pharmacy First – Liberating Capacity. http://psnc.org.uk/dudley-lpc/wp-content/uploads/sites/78/2015/02/Final-V2-Pharmacy-First-Liberating-Capacity-Feb-2015-pdfv.pdf. Accessed 25 Jan 2018.

[29] International Pharmaceutical Federation. An overview of current pharmacy impact on immunization. https://fip.org/files/fip/publications/FIP_report_on_Immunisation.pdf. Accessed 26 Jan 2018.

[30] Wright D, Twigg M, Thornley T (2015). Chronic obstructive pulmonary disease case finding by community pharmacists: a potential cost-effective public health intervention case finding service in England, estimating costs and effects. Int J Pharm Pract.

[31] Pharmaceutical Group of the European Union. Annual Report 2016. http://pgeu.eu/en/library/561:annual-report-2016.html. Accessed 26 Jan 2018.

[32] Rosen B, Waitzberg R, Merkur S (2015). Israel health system review. Health Syst Transit.

[33] State of Israel. Ministry of Health. Procedures for pharmacological management of patients with chronic illness, 2013. https://www.health.gov.il/hozer/mr03_2013.pdf. Accessed 29 Jan 2018. [Hebrew].

[34] Garfinkel D, Mangin D (2010). Feasibility study of systematic approach for discontinuation of multiple medications in older adults. Arch Intern Med.

[35] Gallagher P, O'Mahony D (2008). STOPP (screening tool of older persons’ potentially inappropriate prescriptions): application to acutely ill elderly patients and comparison with beers’ criteria. Age Ageing.

[36] Aparasu RP, Mort JR (2000). Inappropriate prescribing for the elderly: beers criteria-based review. Ann Pharmacother.

[37] State of Israel. Ministry of Health. Ministry of Health’s financial support criteria for the support of pharmaceutical counseling in HMOs. https://www.health.gov.il/Subjects/Finance/Mtmicha/Documents/567152_07082015.pdf . Accessed 26 Apr 2018. [Hebrew].

[38] State of Israel. Ministry of Health. The pharmacy legislation update (pharmacist prescribing), 2014. https://www.health.gov.il/LegislationLibrary/Rokhut30.pdf. Accessed 26 Jan 2018. [Hebrew].

[39] State of Israel. Ministry of Health. HMO financial support of pharmaceutical counseling results: analysis result. 2017. Data on file.

[40] State of Israel. Ministry of Health. Appropriate conditions for simple and moderate unsterile compounding, regulation no. 132, 2014. https://www.health.gov.il/hozer/DR_132.pdf. Accessed 26 Jan 2018. [Hebrew].

[41] State of Israel. Ministry of Health. Appropriate conditions for high risk compounding: sterile and aseptic preparations, regulation no. 135, 2016. https://www.health.gov.il/hozer/DR_135.pdf. Accessed 26 Jan, 2018. [Hebrew].

[42] State of Israel. Ministry of Health. Homeopathic products, regulation no. 10, updated 2014. https://www.health.gov.il/hozer/DR_10.pdf. Accessed 26 Jan 2018. [Hebrew].

[43] Pharmaceutical Association of Israel. Regulation 160: Influenza vaccine administration by a pharmacist in a pharmacy. https://www.pharmacy.org.il/communication/נוהל-160-מתן-חיסון-נגד-שפעת-על-ידי-רוקח-בבי/. Accessed 24 Apr 2018. [Hebrew].

[44] State of Israel. Ministry of Health. Delivery of pharmacy products via messengers or online purchases and emergency dispensing, regulation no. 128, 2014. https://www.health.gov.il/hozer/DR_128.pdf. Accessed 26 Jan 2018. [Hebrew].

[45] State of Israel. Ministry of Health. Qual Assur for private and institutional product importation in accordance to clause 29 of the pharmacist ordinance, regulation no. 129, 2014. https://www.health.gov.il/hozer/DR_129.pdf. Accessed 26 Jan 2018. [Hebrew].

[46] Lomnicky Y, Kenig T, Katzir Z, Hyman A, Tomer G. Improving the quality of pharmaceutical management among diabetic patients using a pharmacist [Abstract]. The Israel National Institute for Health Policy Research. 2014. http://www.israelhpr.org.il/advertising/988.htm. Accessed 26 Jan 2018. [Hebrew].

[47] Brammli-Greenberg S, Shavit O. Pilot study towards an optimal model for pharmaceutical counseling in the Israeli Community Dent Health setting [Abstract]. The Israel National Institute for Health Policy Research. 2012. http://www.israelhpr.org.il/advertising/870.htm. Accessed 26 Jan 2018. [Hebrew].

[48] Triki N (2011). The impact of rationalizing drug utilization on health outcomes. [Dissertation].

[49] Triki N, Guy S, Katz M, Raz M. Proper use of inhaled steroids [Abstract]. The Israel National Institute for Health Policy Research. 2003. http://www.israelhpr.org.il/1058/. Accessed 26 Apr 2018. [Hebrew].

[50] American Pharmacists Association. Principles of Practice for Pharmaceutical Care. https://www.pharmacist.com/principles-practice-pharmaceutical-care. Accessed 25 Apr 2018.

[51] State of Israel. Ministry of Health. Robot at the Hillel Yaffe Medical Center. http://hy.health.gov.il/?CategoryID=214&ArticleID=1785. Accessed 31 Jan 2018. [Hebrew].

[52] State of Israel. Ministry of Health. Pharmacy robot at Shaare Zedek Medical Center. https://www.szmc.org.il/heb/article/news,142/. Accessed 31 Jan 2018. [Hebrew].

[53] State of Israel. Ministry of Health, Guidelines for automated systems and robots utilized for drug distribution and compounding at pharmacies, regulation no. 141, 2015. https://www.chamber.org.il/media/153428/nohal141-17-11-15.pdf. Accessed 31 Jan 2018. [Hebrew].

[54] Koutnik-Fotopoulos E. Automation benefits pharmacies large and small. Pharmacy Times December 1, 2008. http://www.pharmacytimes.com/publications/issue/2008/2008-12/2008-12-5030. Accessed 31 Jan 2018.

[55] Zwaenepoel L, Bilo R, De Boever W, De Vos WM, Reyntens J, Hoorens V, al e (2005). Desire for information about drugs: a survey of the need for information in psychiatric in-patients. Pharm World Sci.

[56] Cordina M, McElnay JC, Hughes CM (1998). Societal perceptions of community pharmaceutical services in Malta. J Clin Pharm Ther.

[57] Bell HM, McElnay JC, Hughes CM (2000). Societal perspectives on the role of the community pharmacist and community based pharmacy services. J Soc Adm Pharm.

[58] Israeli Medical Association. Expanding nurses, pharmacist and other para medical profession authorities – 2005, (Hebrew). https://www.ima.org.il/mainsitenew/ViewCategory.aspx?CategoryId=840. Accessed 12 Aug 2018.

[59] Israeli Medical Association. Position paper concerning pharmacist regulation (prescribing by pharmacist with personal authorization) – 2013 (Hebrew). https://www.ima.org.il/userfiles/image/pharmasistRegulation2013.pdf. Accessed 12 Aug 2018.

[60] Israeli Medical Association. Position paper concerning proposed amendment to pharmacy ordinance (No 23) 2014 – regarding emergency dispensing by a pharmacist with a physician's prescriptions and without personal authorization. https://www.ima.org.il/userfiles/image/nipukTrufaEmergencyHariStand2016.pdf. Accessed 12 Aug 2018.

[61] Vaknin S, Abadi-Korek Y, Marom E. Polypharmacy drug management in patient-pharmacists meeting at the community – the pharmacist’s viewpoint [Abstract]. The Israel National Institute for Health Policy Research. 2012. http://www.israelhpr.org.il/advertising/831.htm. Accessed 26 Jan 2018. [Hebrew].

[62] State of Israel. Ministry of Health, Defining chronic illnesses for medication purchase limit, 2015. https://www.health.gov.il/hozer/sbn07_2015.pdf. Accessed 26 Jan 2018. [Hebrew].

[63] National Institutes of Health. National Heart, Blood, and Lung Institute. Guidelines for the Diagnosis and Management of Asthma 2007. https://www.nhlbi.nih.gov/files/docs/guidelines/asthsumm.pdf. Accessed 25 Apr 2018.

[64] Global Initiative for Chronic Obstructive Lung Disease. Pocket Guide to COPD Diagnosis, Management, and Prevention http://goldcopd.org/wp-content/uploads/2016/12/wms-GOLD-2017-Pocket-Guide.pdf. Accessed 27 June 2017.

