# Supplementary material for: Clinical and other specialty services offered by pharmacists in the community: the international arena and Israel
Source: Isr J Health Policy Res. 2018 Dec 1;7:59. doi: 10.1186/s13584-018-0251-y (PMC6271400; doi:10.1186/s13584-018-0251-y)
Supplement: Supplementary file 1 — Examples of Clinical and other Specialty Services Provided by Pharmacists in the Community Internationally. (DOCX 35 kb) [file 13584_2018_251_MOESM1_ESM.docx]

**Additional file**

**Examples of Clinical and other Specialty Services Provided by Pharmacists in the Community Internationally**

***Transition of Care and Medication Therapy Management (MTM)***

Pharmaceutical care must be practiced in a manner that meets the patient’s individual needs. Failure to do so can results in suboptimal drug therapy or in adverse treatment outcomes. A report by the Pharmaceutical Group of the European Union (PGEU) [1] estimates that 90% of adverse drug reactions (ADRs; noxious or unintended reactions that are caused by taking normal doses of a drug [2]) seen in the elderly population are preventable. Although in other population groups the percentage might be lower, the occurrence of ADRs is also of concern for them as well.

Adverse drug events (ADEs) are those events occurring at a time that a drug is used, but are not necessarily caused by the drug [2]. They can occur throughout the healthcare delivery process; however, the likelihood of ADEs occurring increases during transitions of care (e.g., discharge from a hospital to a nursing home or patients’ move from one health care provider or setting to another)[3].

Within the UK there are several programs that are aimed at extending the role of the community pharmacist to include provision of services at time of changes in drug therapy, such as at hospital discharge. [4] In the “Help for HARRY (**H**igh number/high risk medicines; **A**dherence / medicine knowledge is poor; **R**evised medications; **R**ecently readmitted; **Y**oung no more) discharge referral service”, a referral is made to the patient’s chosen community pharmacy where advanced services (Medicines Use Review [MUR] or New Medicine Service [NMS]) are provided. This service, which was initiated around 2013, is offered to individuals identified as being at high risk due to factors noted in the aforementioned acronym. The MUR is a comprehensive drug review that includes all pharmaceuticals and nutraceuticals taken by the patient. This allows the pharmacist to advise the patient on medication management and on all other necessary adjustments. The service can be offered in a consultation room as well as an over-the-phone conversation. The NMS service is offered to patients recently discharged with a newly prescribed medication in a chronic illness category (asthma, chronic obstructive pulmonary disease [COPD], type 2 diabetes, or those requiring anticoagulant/antiplatelet therapy or antihypertensive medications). The pharmacist provides advice regarding the new medication and tracks the patient's adherence within the first 21 days in the community and suggests ways to increase compliance. An analysis of 75 completed MURs reported that 22% of the patients had been taking incorrect dosages of medication, 33% needed a referral to a general practitioner and that 17% were using old medications.

On the Isle of Wight, a similar program (“Reablement Service”) is offered [5]. Prior to hospital discharge, patients identified as being at a higher risk of readmission, due to an inability to manage their medications, are assessed by a hospital pharmacist. A referral to a community pharmacist can be made and a home visit arranged. During such visits a full medication review is carried out to make sure the right medications are being taken, any discontinued medications are safely disposed of, and if any support services such as home delivery and refills are needed, these are arranged. The service has run for 3 years and was found to reduce readmissions, shortened hospital stays, and released over £800,000 worth of healthcare for local patients.

Twigg et al reported on another service offered in England [6] in which patients over the age of 65 who were prescribed 4 or more medications were invited to participate in a service offered in 25 community pharmacies. The pharmacist evaluated the medications’ appropriateness using standard criteria, held regular consultations with the patients and discussed risk of falls, pain management, adherence and general health. An analysis of the first 6 months of the service found that 620 patients were recruited with 441 (71%) completing the 6-month study period. During that time, pharmacists made 142 recommendations to prescribers for 110 patients, largely centered on potentially inappropriate prescribing of non-steroidal anti-inflammatory drugs (NSAIDs), proton pump inhibitors (PPIs) or duplication of therapy. At follow-up, there was a significant decrease in the total number of falls (mean -0.116 [-0.217--0.014]) and a significant increase in adherence to medications (mean difference in Morisky Measure of Adherence Scale-8: 0.513 [0.337-0.689]) and quality of life. Cost per quality-adjusted life year estimates ranged from £11,885 to £32,466 depending on the assumptions made. The authors concluded that by focusing on patients over the age of 65 years with 4 or more medications, community pharmacists can improve adherence to medications and patients’ quality of life.

In a similar concept to the NMS service yet albeit in a different continent, in a study in the US that was conducted by [Twigg](https://www.ncbi.nlm.nih.gov/pubmed/?term=Twigg%20G%5BAuthor%5D&cauthor=true&cauthor_uid=28483010) et al. [7], a multidisciplinary team led by a community pharmacist (certified as a diabetes educator) provided MTM to geriatric patients (65+), high-risk, diabetic Medicare beneficiaries. The MTM service included a comprehensive medication review as well as self-management education. The study cohort saw a 90% reduction in the number of patients with an A1C > 9% compared with baseline. Adverse drug reactions were reduced by 49%. From the above mentioned services, one can realize the significant effect of the intervention by community pharmacists in diabetes management.

***Medications Adherence and Compliance***

According to a review published in 2016 [8], which included observational studies published between 2000 and 2015, the median (with interquartile range [IQR]) prevalence of ADR-related hospitalization in developed and developing countries was 6.3 % (3.3-11.0) and 5.5 % (1.1-16.9), respectively. The median proportions of preventable ADRs in developed and developing countries were 71.7 % (62.3-80.0) and 59.6 % (51.5-79.6), respectively. Similarly, the median proportions of ADRs resulting in mortality in developed and developing countries were 1.7 % (0.7-4.8) and 1.8 % (0.8-8.0), respectively. Furthermore, according to the recent report by the PGEU [1] 69% of medication-related hospital admissions are due to poor drug adherence. As such, raising the patient's awareness and dedication to the medical treatment can help ameliorate this problem. In one prospective study [9] conducted at an independent community pharmacy in the Midwest US, patients with a proportion of days covered (PDC; a measure of the percent of time that patients have their medication on hand, available for use) for their oral anti-diabetic medications of less than 80% were contacted by telephone and interviewed/counseled by a pharmacist. The change in PDC 120 days post-interview was assessed to determine the change in adherence rates. The investigators reported that the average baseline PDC (*n* = 25) was 65.2%. The average 120-day post interview PDC rose to 78.7%, a 13.5% increase (*p* < 0.002). The authors noted that this is likely to indicate more regular medication-taking at home. This illustrates the potential benefit that pharmacists counseling may have on patients’ compliance with their medications.

Abughosh et al [10] reported on a study to examine the effect of a brief pharmacist telephone intervention in identifying adherence barriers and improving adherence to angiotensin converting enzyme inhibitors (ACEIs) and angiotensin receptor blockers (ARBs) among non-adherent patients with comorbid hypertension (HTN) and diabetes mellitus (DM) who were enrolled in a Medicare Advantage plan in Texas. The telephone intervention was carried out among 87 patients randomly selected from the patients identified who were non-adherent to their ACEI/ARB medications. Patients who were not contacted were in the control group. The investigators reported that the intervention was a significant predictor of better adherence in the linear regression model after adjusting all the other baseline covariates (β = 0.3182, 95% CI = 0.19-0.38, *P* < 0.001). The investigators concluded that the brief telephone intervention resulted in significantly better PDCs during the 6 months following the intervention as well as lower discontinuation rates among a group of non-adherent patients with comorbid HTN and DM.

Kovačević et al described a study [11] that evaluated the impact of pharmacist-delivered counselling on patients’ knowledge and beliefs about their medications, adherence level, and asthma control. The study, was conducted in Serbian community pharmacies, in which pharmacists delivered a counseling session on asthma. Each session lasted 30 minutes on average. Statistically significant improvement was found after 3 months in patients knowledge of asthma and its medications, their attitude towards medications (decrease in harm, overuse and concern; increase in necessity score), asthma control score (increased from 19 to 20, p < 0.05) and level of adherence (MMAS-8 score decreased from 3 to 2 p < 0.05). Better asthma control was achieved in 60% of patients. Sixteen patients (18%) were transferred from poor to well-controlled asthma, implying no need for patients' referral to the doctor and no additional cost for the health system.

Single-intervention research studies among asthmatics have been conducted in several countries (Schulz et al. in Germany [12], [García-Cárdenas](https://www.ncbi.nlm.nih.gov/pubmed/?term=Garc%C3%ADa-C%C3%A1rdenas%20V%5BAuthor%5D&cauthor=true&cauthor_uid=23810267) et al. in Spain [13], and [Wong](https://www.ncbi.nlm.nih.gov/pubmed/?term=Wong%20LY%5BAuthor%5D&cauthor=true&cauthor_uid=28472499) et al. in Malaysia^[[1]](#footnote-1)^ [14]) following roughly the same structure. In each case, asthmatic patients with different degrees of disease severity were randomly assigned into an intervention groups and control groups. The control groups continued ordinary patient-pharmacist communication, while the intervention groups received a one-time pharmacy management service. The management included guidance on proper inhaler use (using "Teach-Back"^[[2]](#footnote-2)^ method) and explanation regarding the illness. In addition, attention was given to the patient's personal questions and needs. In both the Spanish and the German studies, 90% of the patients in the intervention group reported stable asthma, thrice the numbers seen in the control groups. Six months after the start of the study, there was still a significant rise in proper use among patients who underwent intervention, and despite the intervention being a one-time event, there was also a 40% rise in adherence to medications compared to the control group.

Considering prolonged chronic-illnesses, in a study conducted by Närhi et al.[15], 31 patients suffering from unstable asthma^[[3]](#footnote-3)^ consulted with physicians, nurses, and community-pharmacists periodically over the period of 1 year. Twenty-eight patients finished the study, visiting their designated pharmacist at least once every 3 months. On average, 3 out of 5 symptoms of the disease improved significantly in each patient. The number of patients with severe shortness of breath decreased from 7 to 4, fewer patients required steroid treatment following the study, and more than half (57%) "stepped down" in the degree of classification of asthma.

A prospective intervention study by Stuurman-Bieze et al [16] included 1,002 patients from 9 Dutch community pharmacies initiating lipid lowering therapy. The investigators assessed the effectiveness of the proactive pharmaceutical care intervention (Medication Monitoring and Optimization; MeMO) plan, that continued for a year, on the discontinuation rate and patient adherence as compared to a historical control group. The MeMO program started with structured counseling sessions and follow-up of chronic therapies. The process was followed by a continuous phase in which patients' therapy adherence was monitored on a monthly basis, using standardized search algorithms in the pharmacy database. When the algorithm detected discontinuation of therapy, tailored interventions were used to improve adherence and optimize pharmacotherapy. The investigators found that drug discontinuation rates in the first year after initiation were 13.6% for the intervention group and 25.9% in the usual care (control) group; continued but non-adherent use was 3.2% and 7.6% in these groups, respectively, and that patients in the MeMO program had a decreased risk of discontinuing medications of 51% (95% confidence interval [34%-63%]).

Apart from “targeted interventions", another type of service offered to chronic patients, is Appointment Based Model (ABM) [17]. Under this model, each patient enrolled in the ABM has a designated appointment day to pick up all medications. Pharmacy staff call patients in advance of their appointment to identify any changes to the medications and confirm that each prescription should be refilled. Such a method allows pharmacists to have the medication ready in advance of an appointment, as well as provide maximum care and attention for the patient's needs and concerns with minimum waiting time. In a study by Holdford and Inocencio [18], the investigators examined the appointment-based medication synchronization (ABMS) method on medication adherence and persistence with chronic medications. In this program, which was implemented in rural pharmacies in the Midwestern US, patients assigned to the intervention group met with a pharmacist to solve medication-related problems. Likewise, their medications were synchronized to be dispensed on a single day of the month. Compared with control patients, those in the ABMS group had 3.4 to 6.1 times greater odds of adherence compared with control patients. Control patients were 52% to 73% more likely to stop taking their chronic medications over 1 year.

***Urgent Care and Preventative Services***

In the UK, pharmacists at 192 community pharmacies were trained to provide a minor ailment service under a campaign called "The Pharmacy First" [19]. The goal of this service was to provide first line urgent care for patients with minor ailments. Key findings of the 3-month pilot phase of the program were that 25,956 minor ailment service (MAS) consultations were undertaken, 79% of all MAS consultations shown to shift workload from local general practices (GP) to community pharmacies, 2 hours per week of GP appointments were “liberated” per 5900 registered patients, 83% of the patients surveyed following a MAS consultation would recommend the service.

According to a 2016 report by the International Pharmaceutical Federation [20], as of 2016, 13 countries authorized pharmacists to administer vaccines. These included Argentina, Australia, Canada, Costa Rica, Denmark, Ireland, New Zealand, Philippines, Portugal, South Africa, Switzerland, UK, and US. Notably, only 45 of the 137 surveyed FIP member organizations responded to the survey; thus, it is possible that more countries authorize vaccination by pharmacists then those listed. A study by Drozd et al. [21], evaluated changes in influenza immunization rates in the US between 2003 and 2013. The researchers found that as states moved to allow pharmacists to administer influenza immunizations, the odds that an adult received an influenza immunization rose. The average percentage of people receiving influenza immunizations rose from 32.2% in 2003 to 40.3% in 2013.

Consultation Services’ as Means of Decreasing Hospitalizations and Rehabilitation Programs

A meta-analysis conducted by the Cochrane group [22], examined the influence of "non-dispensing" pharmacy roles of 40 pharmacists to over 16,000 patients in several countries. The analysis also included 7 studies where the pharmacist intervention was targeted at other health professionals (and not directly to patients). This meta-analysis found that when a pharmacist provides pharmaceutical care and counseling, the number of appointments a patient requires from a medical staff (including the pharmacist) increases slightly; however, it lowered the number of hospitalizations as well as emergency room (ER) visits significantly.

In addition to the services examined by the Cochrane group, pharmacies also provide care to patients from specific weak populations. These include drug abusers who partake in a methadone treatment program to assist in rehabilitation. Some specialized pharmacies only distribute the methadone, while in others the methadone is taken in the pharmacy under the pharmacist’s supervision. Such service may be offered in all community pharmacies in Scotland [23], with 72% providing the drug to consumers, and 65.1% of the consumers using the methadone under pharmacist supervision. The service as well as the supervision assists greatly in supporting the addict in his or her rehabilitation process.

***Health Education, Health Screening and Early Detection of Disease***

In 2012-2013 a pilot program in the UK offered a service in community pharmacies to screen patients potentially at risk of COPD. [24] The program was aimed at early detection (through “case-finding” consisting of screening people at risk of COPD using a symptom questionnaire and spirometry test to evaluate lung function) of COPD. Smoking cessation was offered to all smokers identified as potentially having undiagnosed COPD. Cost and effects of the service were estimated. Over the course of 9 month, 21 community pharmacies screened 238 patients. One hundred thirty-five patients were identified with potentially undiagnosed COPD; 88 were smokers. Smoking cessation initiation provided a projected gain of 38.62 life years, 19.92 quality-adjusted life years and a cost saving of £392.67 per patient screened.

Throughout Europe, blood pressure screening is conducted in community pharmacies in 22 countries, BMI assessments in 23 countries, and blood glucose and cholesterol levels assessments in 20 countries [25]. Likewise, bowel cancer screening programs are provided through pharmacies in Italy, Spain, and Switzerland. Examples of other chronic disease management programs and educational programs conducted in some European community pharmacies include diabetes management (15 countries), asthma management (14 countries), hypertension management (14 countries), and smoking cessation (20 countries).

***References***

1. Pharmaceutical Group of European Union. Overview of Community Pharmacy Services in Europe (PGEU). <https://www.oecd.org/els/health-systems/Item-2b-Overview-Community-Pharmacy-Services-Svarcaite%20.pdf>. Accessed 22 Jan 2018.
2. World Health Organization. Definitions. [http://www.who.int/medicines/areas/quality_safety/safety_efficacy/trainingcourses/definitions.pdf. Accessed 24 Apr 2018](http://www.who.int/medicines/areas/quality_safety/safety_efficacy/trainingcourses/definitions.pdf.%20Accessed%2024%20Apr%202018).
3. U.S. Department of Health and Human Services. Office of Disease Prevention and Health Promotion. National Action Plan for Adverse Drug Event Prevention. <https://health.gov/hcq/pdfs/ADE-Action-Plan-508c.pdf>. Accessed 23 Jan 2018.
4. NHS England. Quick Guide. Extending the Role of Community Pharmacy in Urgent Care. <https://www.england.nhs.uk/commissioning/wp-content/uploads/sites/12/2015/11/quick-guid-comm-pharm-urgent-care.pdf>. Accessed 23 Jan 2018.
5. NHS Isle of Wight, Getting medicines right in hospital and at home, <http://www.iow.nhs.uk/default.aspx.locid-02gnew08v.Lang-EN.htm>. Accessed 23 Jan 2018.
6. [Twigg MJ](https://www.ncbi.nlm.nih.gov/pubmed/?term=Twigg%20MJ%5BAuthor%5D&cauthor=true&cauthor_uid=25847545), Wright D, Barton GR, Thornley T, Kerr C. The Four or more medicines (FOMM) support service: results from an evaluation of a new community pharmacy service aimed at over-65s. [*Int J Pharm Pract.*](https://www.ncbi.nlm.nih.gov/pubmed/25847545) 2015;23(6):407-14.
7. [Twigg G](https://www.ncbi.nlm.nih.gov/pubmed/?term=Twigg%20G%5BAuthor%5D&cauthor=true&cauthor_uid=28483010), Motsko J, Thomas J, David T. Pharmacist-managed diabetes center interventions ensure quality and safety in elderly patients. *Consult Pharm.* 2017;32(5):299-310.
8. [Angamo MT](https://www.ncbi.nlm.nih.gov/pubmed/?term=Angamo%20MT%5BAuthor%5D&cauthor=true&cauthor_uid=27449638), [Chalmers L](https://www.ncbi.nlm.nih.gov/pubmed/?term=Chalmers%20L%5BAuthor%5D&cauthor=true&cauthor_uid=27449638), [Curtain CM](https://www.ncbi.nlm.nih.gov/pubmed/?term=Curtain%20CM%5BAuthor%5D&cauthor=true&cauthor_uid=27449638), [Bereznicki LR](https://www.ncbi.nlm.nih.gov/pubmed/?term=Bereznicki%20LR%5BAuthor%5D&cauthor=true&cauthor_uid=27449638). Adverse-drug-reaction-related hospitalisations in developed and developing countries: a review of prevalence and contributing factors. [*Drug Saf*.](https://www.ncbi.nlm.nih.gov/pubmed/27449638) 2016;39(9):847-57. doi: 10.1007/s40264-016-0444-7.
9. [Singleton](https://www.ncbi.nlm.nih.gov/pubmed/?term=Singleton%20J%5BAuthor%5D&cauthor=true&cauthor_uid=29048348) J, Veach S, Catney C, Witry M. Analysis of a community pharmacy intervention to improve low adherence rates to oral diabetes medications. *Pharmacy*. 2017; 5(4): 58.
10. [Abughosh](https://www.jmcp.org/author/Abughosh%2C+Susan+M) SM, [Wang](https://www.jmcp.org/author/Wang%2C+Xin) X, [Serna](https://www.jmcp.org/author/Serna%2C+Omar) O, [Henges](https://www.jmcp.org/author/Henges%2C+Chris) C, [Masilamani](https://www.jmcp.org/author/Masilamani%2C+Santhi) S, [Essien](https://www.jmcp.org/author/James+Essien%2C+Ekere) EJ,et al. A pharmacist telephone intervention to identify adherence barriers and improve adherence among nonadherent patients with comorbid hypertension and diabetes in a Medicare Advantage Plan. *J Manag Care Spec Pharm.* 2016;22(1):63-73.
11. [Kovačević M](https://www.ncbi.nlm.nih.gov/pubmed/?term=Kova%C4%8Devi%C4%87%20M%5BAuthor%5D&cauthor=true&cauthor_uid=28778676), [Ćulafić M](https://www.ncbi.nlm.nih.gov/pubmed/?term=%C4%86ulafi%C4%87%20M%5BAuthor%5D&cauthor=true&cauthor_uid=28778676), [Jovanović M](https://www.ncbi.nlm.nih.gov/pubmed/?term=Jovanovi%C4%87%20M%5BAuthor%5D&cauthor=true&cauthor_uid=28778676), [Vučićević K](https://www.ncbi.nlm.nih.gov/pubmed/?term=Vu%C4%8Di%C4%87evi%C4%87%20K%5BAuthor%5D&cauthor=true&cauthor_uid=28778676), [Kovačević SV](https://www.ncbi.nlm.nih.gov/pubmed/?term=Kova%C4%8Devi%C4%87%20SV%5BAuthor%5D&cauthor=true&cauthor_uid=28778676), [Miljković B](https://www.ncbi.nlm.nih.gov/pubmed/?term=Miljkovi%C4%87%20B%5BAuthor%5D&cauthor=true&cauthor_uid=28778676). Impact of community pharmacists' interventions on asthma self-management care*.* [*Res Social Adm Pharm.*](https://www.ncbi.nlm.nih.gov/pubmed/28778676) 2017: https://doi.org/10.1016/j.sapharm.2017.07.007.
12. Schulz M, Verheyen F, [Mühlig S,](https://accp1.onlinelibrary.wiley.com/action/doSearch?ContribAuthorStored=M%C3%BChlig%2C+Stephan) [Müller JM,](https://accp1.onlinelibrary.wiley.com/action/doSearch?ContribAuthorStored=M%C3%BCller%2C+J%C3%B6rg+Michael) [Mühlbauer K,](https://accp1.onlinelibrary.wiley.com/action/doSearch?ContribAuthorStored=M%C3%BChlbauer%2C+Katrin) [Knop‐Schneickert](https://accp1.onlinelibrary.wiley.com/action/doSearch?ContribAuthorStored=Knop-Schneickert%2C+Elke) E, et al. Pharmaceutical care services for asthma patients: A controlled intervention study. *Journal of Clinical Pharmacology.* 2001;41(6):668-76. DOI: 10.1177/00912700122010438
13. [García-Cárdenas V,](https://www.ncbi.nlm.nih.gov/pubmed/?term=Garc%C3%ADa-C%C3%A1rdenas%20V%5BAuthor%5D&cauthor=true&cauthor_uid=23810267) [Sabater-Hernández D](https://www.ncbi.nlm.nih.gov/pubmed/?term=Sabater-Hern%C3%A1ndez%20D%5BAuthor%5D&cauthor=true&cauthor_uid=23810267), [Kenny P](https://www.ncbi.nlm.nih.gov/pubmed/?term=Kenny%20P%5BAuthor%5D&cauthor=true&cauthor_uid=23810267), [Martínez-Martínez F](https://www.ncbi.nlm.nih.gov/pubmed/?term=Mart%C3%ADnez-Mart%C3%ADnez%20F%5BAuthor%5D&cauthor=true&cauthor_uid=23810267), [Faus MJ](https://www.ncbi.nlm.nih.gov/pubmed/?term=Faus%20MJ%5BAuthor%5D&cauthor=true&cauthor_uid=23810267), [Benrimoj SI](https://www.ncbi.nlm.nih.gov/pubmed/?term=Benrimoj%20SI%5BAuthor%5D&cauthor=true&cauthor_uid=23810267). Effect of a pharmacist intervention on asthma control. A cluster randomized trial, respiratory medicine. 2013 ;107(9):1346-55. doi: 10.1016/j.rmed.2013.05.014.
14. [Wong LY](https://www.ncbi.nlm.nih.gov/pubmed/?term=Wong%20LY%5BAuthor%5D&cauthor=true&cauthor_uid=28472499), [Chua SS](https://www.ncbi.nlm.nih.gov/pubmed/?term=Chua%20SS%5BAuthor%5D&cauthor=true&cauthor_uid=28472499), [Husin AR](https://www.ncbi.nlm.nih.gov/pubmed/?term=Husin%20AR%5BAuthor%5D&cauthor=true&cauthor_uid=28472499), [Arshad H](https://www.ncbi.nlm.nih.gov/pubmed/?term=Arshad%20H%5BAuthor%5D&cauthor=true&cauthor_uid=28472499). A pharmacy management service for adults with asthma: a cluster randomized controlled trial. *Fam Pract.* 2017;34(5):564-573. doi: 10.1093/fampra/cmx028.
15. Närhi U, Airaksinen M, Tanskanen P, Erlund H. Therapeutic outcomes monitoring by community pharmacists for improving clinical outcomes in asthma. *J Clin Pharm Ther.* 2000;25(3):177-83.
16. Stuurman-Bieze AGG, Hiddink EG, van Boven JFM, Vegter S. Proactive pharmaceutical care interventions improve patients’ adherence to lipid-lowering medication. *The Annals of Pharmacotherapy.* 2013;47(11):1448 –1456.
17. APhA Foundation. Pharmacy’s Appointment Based Model (ABM). <https://www.aphafoundation.org/appointment-based-model>. Accessed 2 Feb 2018.
18. Holdford DA, Inocencio TJ. Adherence and persistence associated with an appointment-based medication synchronization program. *J Am Pharm Assoc.* 2013;53:576–583.
19. NHS England. Pharmacy First – Liberating Capacity. <http://psnc.org.uk/dudley-lpc/wp-content/uploads/sites/78/2015/02/Final-V2-Pharmacy-First-Liberating-Capacity-Feb-2015-pdfv.pdf>. Accessed 25 Jan 2018.
20. International Pharmaceutical Federation. An overview of current pharmacy impact on immunization. <https://fip.org/files/fip/publications/FIP_report_on_Immunisation.pdf>. Accessed 26 Jan 2018.
21. Drozd EM, Miller L, Johnsrud M. Impact of pharmacist immunization authority on seasonal influenza immunization rates across states. *Clinical Theraputics.* 2017;39:1563–1580.
22. Nkansah N, Mostovetsky O, Yu C, Chheng T, Beney J, Bond CM, Bero L. Effect of outpatient pharmacists' non-dispensing roles on patient outcomes and prescribing patterns. [*Cochrane Database Syst Rev.*](https://www.ncbi.nlm.nih.gov/pubmed/20614422) 2010;(7):CD000336. doi: 10.1002/14651858.CD000336.pub2.
23. [Matheson C](https://www.ncbi.nlm.nih.gov/pubmed/?term=Matheson%20C%5BAuthor%5D&cauthor=true&cauthor_uid=12410781), Community pharmacy services for drug misusers in Scotland: what difference does 5 years make? *Addiction.* 2002;97(11):1405-11. DOI: 10.1046/j.1360-0443.2002.00241.x.
24. Wright D, Twigg M, Thornley T. Chronic obstructive pulmonary disease case finding by community pharmacists: a potential cost-effective public health intervention) case finding service in England, estimating costs and effects. *International Journal of Pharmacy Practice.* 2015;23:83–85.

[25]. Pharmaceutical Group of the European Union. Annual Report 2016. <http://pgeu.eu/en/library/561:annual-report-2016.html>. Accessed 26 Jan 2018.

1. Malaysia operates a different health-care structure from the western countries. The pharmacists in this experiment were government clinic pharmacies. For the purpose of this article it was decided to include them under community pharmacies. [↑](#footnote-ref-1)
2. The "Teach-Back" system is an educational method commonly used in the medical world. In this method a medical-professional teaches a patient how to use a device, or medication, and then asks the patient to show him or her how to use the device, as if he or she is the one teaching the medical staff. This method has shown to be very effective in implementing understanding in the patient population. [↑](#footnote-ref-2)
3. Unstable asthma was defined by the research as at least 1 asthma attack a week for a month. [↑](#footnote-ref-3)
